# Modelling of the dilated sagittal sinuses found in multiple sclerosis suggests increased wall stiffness may be a contributing factor

**DOI:** 10.1038/s41598-022-21810-3

**Published:** 2022-10-20

**Authors:** Grant Alexander Bateman, Jeannette Lechner-Scott, Alexander Robert Bateman

**Affiliations:** 1 Department of Medical Imaging, John Hunter Hospital, Newcastle Region Mail Center, Locked Bag 1, Newcastle, NSW 2310 Australia; 2 Faculty of Health, Newcastle University, Callaghan Campus, Newcastle, NSW Australia; 3 Department of Neurology, John Hunter Hospital, Newcastle, NSW Australia; 4 Hunter Medical Research Institute, Newcastle, NSW Australia; 5 School of Mechanical Engineering, University of NSW, Sydney, NSW Australia

**Keywords:** Neurology, Multiple sclerosis

## Abstract

The cross-sectional area of the superior sagittal sinus (SSS) is larger in multiple sclerosis than normal and correlates with disease severity and progression. The sinus could be enlarged due to a decrease in the pressure difference between the lumen and the subarachnoid space, an increase in wall thickness or increased wall stiffness. The cross-sectional area of the SSS and straight sinus (ST) were measured in 103 patients with multiple sclerosis and compared to 50 controls. The cross-sectional area of the SSS and ST were increased by 20% and 13% compared to the controls (*p* = 0.005 and 0.02 respectively). The deflection of the wall of the sinus was estimated. The change in pressure gradient, wall thickness or elastic modulus between groups was calculated by modelling the walls as simply supported beams. To account for these findings, the modelling suggests either a 70% reduction in transmural venous pressure or a 2.4 fold increase in SSS wall stiffness plus an 11% increase in wall thickness or a combination of changes. An increase in sinus pressure, although the most straight forward possibility to account for the change in sinus size may exist in only a minority of patients. An increase in sinus wall stiffness and thickness may need further investigation.

## Introduction

In multiple sclerosis (MS) the superior sagittal sinus (SSS) cross-sectional area was found to be 16% larger than in matched controls^1^. The sinus size seems to have prognostic significance with larger sinuses correlating with male patients, progressive forms of the disease and worsening outcomes^2^. It was noted that in hydrocephalus and spontaneous intracranial hypotension, the cross-sectional area of the SSS varies with the pressure difference between the sinus and the subarachnoid space (the transmural pressure)^1^. This suggests that the larger sinus size in MS could be due to a decrease in the transmural pressure^3^. An alternative cause of the enlarged sinuses could be an increase in the stiffness of the sinus wall^1^ but which of these alternatives is the more feasible?

**Figure 1:**
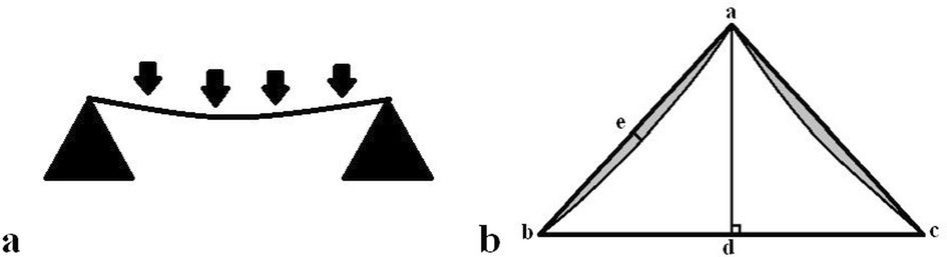
Free wall deflection as a simply supported beam. (**a**) A line drawing showing that a simply supported beam will deflect when under an equally distributed force depending on the size of the force, the beam length, thickness and stiffness. (**b**) A line drawing of an idealized sagittal sinus. Point “a” is at the falx vertex and both “b” and “c” are the bony attachments. If the attachments are fixed in position is can be shown that the two free walls a-b and a-c would be deflected inwards by the transmural pressure similar to the simply supported beam. The difference in the area between the triangle subtended by the vertices and the actual area (shown in grey) is related to the deflection distance at “e” by the length a-b using Eq. (4). The perpendicular height is the line a–d.

The sagittal sinus consists of a venous channel passing through a split in the dura as it passes from the falx cerebri to the skull^4^. The dura at the base of the sinus is attached to the endosteum of the skull and is fixed. The other two walls of the sinus are attached to the falx cerebri and are relatively fixed at this point^1^. Between the three fixed vertices, the two free walls can move. In the transverse sinuses the free walls have been noted to be concave, straight or convex^5^. As previously noted, the walls of the sagittal sinus have also been shown to move depending on the transmural pressure gradient^1^. The dural wall is a viscoelastic structure made up of collagen fibers interspersed with fibroblasts and elastin^6^, so the structural properties of the wall are also important in the degree of deflection. In engineering terms, the degree of sinus wall deflection is similar to the deflection seen in a beam under load (see Fig. 1). Deflection occurs in a beam which is freely supported at both ends due to a force which is equally applied along its length. The size of the deflection depends on the length of the beam, size of the force, the elastic modulus or stiffness and the beam thickness^7^. The elastic modulus can be estimated if the wall length, deflection, wall thickness and the applied force are known^7^. Thus, the purpose of this paper is to use engineering modelling to test the feasibility of whether the sinus walls are altered in their deflection in MS due to an alteration in pressure, wall stiffness or wall thickness.

## Results

The sinus length and cross-sectional area data is summarized in Table 1. The raw data is available online^8^.

**Table 1:** Sagittal and straight sinus measurements.

	Age years	SSS area mm^2^	SSS length mm	SSS height mm	SSS triangle area mm^2^	SSS chord length mm	ST area mm^2^
	**Control**						
Mean	44.9	45.0	12.6	7.9	51.6	10.2	15.7
SD	10.9	16.0	3.1	2.0	22.2	2.2	5.4
n	50						
	**Multiple sclerosis**						
Mean	47.8	54.0	13.5	8.1	56.4	10.6	17.8
SD	13.0	17.6	2.8	1.7	20.7	1.9	5.7
n	103						
MWU	0.28	0.005*	0.08	0.56	0.16	0.23	0.02*

mm, millimeters; mm^2^, millimeters squared; MWU, Mann–Whitney U test; SD, standard deviation; SSS, superior sagittal sinus; ST, straight sinus.

*Significance < 0.05.

The multiple sclerosis patient’s SSS and straight sinus (ST) areas were 20% and 13% larger than the controls (*p* = 0.005 and 0.02 respectively). The sagittal sinus widths and the heights of the attachments from the baseline and vertex for the controls and MS patients were not significantly different. Therefore, the free wall lengths and the areas of the triangles subtended by the attachment points were not significantly different.

### Modelling

The chord length from Table 1 for the controls is 10.2 mm and by subtraction the area of the deflection is the SSS area minus the SSS triangle area divided by 2 or 3.3 mm^2^ per side. Using Eq. (4) this equates to a deflection of 0.48 mm. The normal ICP obtained from the literature is 11.5 mmHg^9^. The normal SSS pressure is 7.5 mmHg at 45 years of age^10^. This gives a normal SSS transmural pressure by subtraction of 4 mmHg, which correlates with the literature^11^. Modelling a strip of sinus wall 1 mm wide (this width was chosen for simplicity but has no bearing on the final outcome) would give an area of the upper surface of 1 mm × the chord length. Placing the area and pressure in Eq. (5) gives an equally applied force of 0.0054 Newtons. The median thickness of the free walls of the posterior portion of the SSS in a human cadaver study was 0.675 mm^12^. If we model a strip of dura 1 mm wide, the moment of inertia of cross-section from Eq. (7) would be 2.56 × 10^–14^ m^4^. Placing these values in Eq. (6) and solving for the elastic modulus gives a result of 6.1 MPa for the controls.

Using the same technique, the deflection in MS is 65% less than in the controls at 0.17 mm. Assuming the elastic modulus and wall thickness are unchanged from the controls, this would give a transmural pressure of 1.2 mmHg or a reduction in pressure of 70% compared to the controls. If we assume that the transmural pressure and wall thickness are unchanged, then the elastic modulus would be increased to 20.1 MPa or 3.3 times normal. If the transmural pressure and elastic modulus were unchanged, then the wall thickness would be 1.0 mm or 1.49 times normal.

## Discussion

The correct engineering model to use in the current study would depend on the type of boundary condition at the attachment points i.e. fixed, roller or pinned^7^. The use of a fixed condition would assume that the angle at the endpoint is zero with respect to the horizontal^7^ (i.e. the attachment does not bend). As the angle at the attachment endpoints are non-zero, the most suitable model would require the equivalent of pinned endpoints i.e. the simply supported beam. The use of a roller condition would yield the same result as the pinned condition in this instance, as the same mathematical assumptions with regard to the beam deflection are used^7^.

The modelling requires an estimate of the force applied across the sinus walls in the controls. Thus, we need to know the CSF and venous pressures. The normal ICP and sinus pressure and therefore the transmural pressures were obtained from the literature.

**Figure 2:**
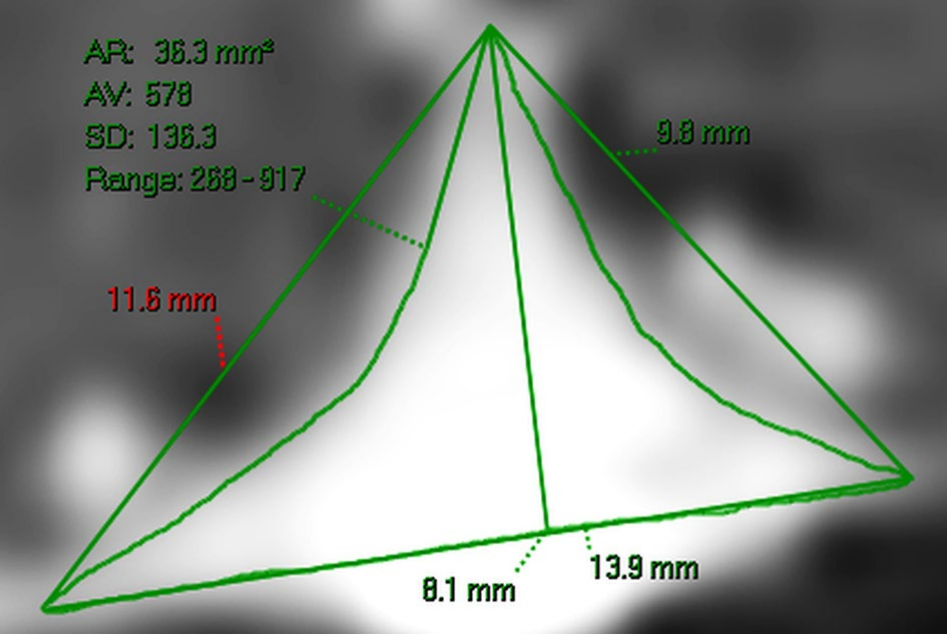
Measurements in a control patient. A reconstruction of the 3DT1 post contrast data from a control patient. The baseline length is 13.9 mm and the perpendicular height is 8.1 mm giving a triangle area of 56.3 mm^2^. The deflection area on each side averages 10 mm^2^ (56.3–36.3/2). The average chord length between the vertex and both base attachments is 10.7 mm using Pythagoras’ theorem. Giving an average deflection distance using Eq. (4) of 1.4 mm which is equivalent to the actual deflection in this case. Note, any sinus area below the baseline is ignored in this modelling study.

The modelling also requires an accurate estimate of the deflection of the walls. It became obvious that direct measurement of the deflection was too inaccurate. The resolution of the post contrast 3D T1 images is 0.85 mm with each pixel being 0.72 mm^2^. The average deflection in the controls was 0.48 mm (half a pixel) but the combined reduction in the sinus area was 6.6 mm^2^ and this represented approximately 9 pixels. Given the attachment points are relatively fixed, the deflection could be estimated from the change in the cross-sectional area. In a cadaver study of adults mean age 39 years, the sagittal sinus just above the Torcular was found to be an isosceles triangle of mean width 11.6 mm and height 8.3 mm with a 48 mm^2^ cross-sectional area and a calculated average free wall length of 10.1 mm^13^. These measurements are very similar to the mean width, height, triangle area and free wall length found in the present study, with the difference between the cadavers and control patients being 8%, 5%, 7% and 1% respectively. Sensitivity analysis suggests an 8% error in either the area or length measurement would have a minimal effect on the final outcome of this study. There is no transmural pressure gradient in a cadaver, meaning the stress free state for the normal sinus walls is for them to be straight. Thus, they were compared to the calculated triangle sinus area. The length measurements in MS were not significantly different to the controls, indicating multiple sclerosis is unlikely to alter the fixed point positions. As a pressure gradient is equivalent to a force which is equally applied in all directions, the expected deflection when compared to the original straight wall position should approximate a circle segment. According to Laplace’s law, when a pressure is applied to a thin membrane, the volume change will be accommodated by the smallest possible change in membrane area because this would give the minimum energy state. The smallest surface area for a given volume is always a circle segment. The same mechanism underlies why cerebral aneurysms develop as circle segments^14^. The deflection as seen in Fig. 2 was calculated from the area between the free wall or chord and the curved line using the circle Eq. (4).

In the controls, a small smooth deflection averaging 0.48 mm develops from a 4 mmHg pressure gradient, giving a Young’s modulus averaging 6.1 MPa. The dural elastic modulus has been measured in humans using fresh samples with the values varying from 29.4 MPa^15^, 44 MPa^16^, up to 61 MPa^17^. The dura from these studies comes from various regions of the skull and it is possible that the differing regions may be optimised for varying degrees of absolute strength rather than flexibility. Recently, Young’s modulus of the sagittal sinus in the mediolateral direction has been directly measured in humans and was found to be 5.2 MPa at a strain of approximately 1%^18^ which is similar to our findings. In the controls the deflected arc length was 0.6% longer than the resting chord length giving an average strain similar to^18^. The wall data from this paper were derived from the frontal, parietal and occipital regions of the sinus. When the data were restricted to the occipital region only (to better match our site of estimation) the 8 samples averaged 6.3 MPa (personal correspondence with Dr Mulvihill), which is an even closer match to our data. In pigs, the dura over the inner table of the skull has been measured to be between 8 and 16 MPa^19^ i.e. the stiffness is somewhat less than that in humans. In pigs, the longitudinal and circumferential SSS stiffness within the occipital region has been measured and found to be 58.1 ± 17.2 MPa longitudinally with the circumferential figure being 3.0 ± 0.7 MPa^19^. The circumferential stiffness in pigs compares to the circumferential stiffness of 6.1 MPa we estimated, again suggesting human dura is somewhat stiffer than porcine. The difference between the longitudinal and circumferential stiffness in the SSS is due to the collagen alignment. The fraction of collagen within the pig sinus wall is 84%. The collagen is randomly directed in that portion of the sinus adjacent to the bone but is longitudinally directed in those portions adjacent to the subarachnoid space^19^. This suggests the free walls of the sinus are designed to be stiff in the longitudinal direction but flexible at 90 degrees to this direction.

The simplest explanation for the 20% larger SSS area in MS, is an increase in sinus pressure compared to the subarachnoid pressure i.e. a reduced transmural pressure. The normal ICP in middle age is 11.5 mmHg^9^, the ICP measured at lumbar puncture in 32 MS patients was 12.9 ± 3.3 mmHg (1.4 mmHg higher), with the MS ICP being identical to normal pressure hydrocephalus patients^20^. Normal pressure hydrocephalus patients are known to have mildly elevated ICPs which are still within the normal range^21^. Thus, in order for the transmural pressure to be normal, the absolute sagittal sinus pressure would also need to be elevated by 1.4 mmHg to match the ICP in MS. The target sinus pressure to completely explain the sinus wall deflection would be an average transmural pressure reduced by 2.8 mmHg to correspond to the 70% reduction. Thus the average absolute SSS pressure would need to rise by 4.2 mmHg (once we take into account the increased ICP). The required pressure increase could come from the neck vessels. Zamboni et al. first suggested neck vessel stenoses may be associated with MS in 2008, calling the condition chronic cerebrospinal venous insufficiency (CCSVI). They found 91% of MS patients had a jugular vein stenosis, with the average pressure gradient across the stenoses being 1.8 cmH_2_O^22^ or 1.3 mmHg. However, other authors have raised doubts about the significance of these findings, noting that although a > 50% stenosis of a major vein occurs in 74% of MS patients, it also occurs in 66% of MS patient’s siblings and 70% of unrelated controls^23^. Despite this, advocates of CCSVI maintain that the stenoses are hemodynamically significant because they alter the flow patterns^22^. The hemodynamic significance we are interested in, is whether the stenoses generate a pressure gradient between the jugular bulb and the right heart. In Zamboni et al.’s original paper the answer to that question was no. In MS the pressure gradient between the jugular vein above the stenosis and the superior vena cava was 1 cmH_2_O or 0.74 mmHg^22^. The normal pressure gradient between these two sites is also 1 cmH_2_O^24^ i.e. identical. How can this be? The answer is that the resistance between the jugular bulbs and the right atrium is very low. This is because of the excellent collateral flow afforded by the vertebral veins and the paravertebral venous plexus. Gadda et al. note, “the venous outflow system is quite robust in response to a single vessel closure, as a single path can be replaced by an alternative route”^25^. Indeed, in order for a significant rise in sinus pressure to occur, a total occlusion of both jugular veins must occur^25^. Bilateral total jugular occlusion can occur but does not appear to be a common occurrence in MS^23^. Therefore, we would expect only a small number of patients could have their wall deflection explained by venous neck pressure alone. Zamboni’s original manometry study may have lacked the resolution to find a small pressure rise. We have scoured the CCSVI literature to find any other evidence of a measured pressure increase across the neck veins, which also takes into account the collateral pathways but have found only one other paper. Beggs et al. utilized neck plethysmography and found a total outflow resistance increase of 64% which would increase the jugular bulb pressure by 0.47 mmHg given the normal pressure gradient of 0.74 mmHg. Thus, advocates of CCSVI may be correct in suggesting there is a significant elevation in the neck vein pressure in MS but this probably only occurs in a small percentage of patients. This realization may shed some light onto why the original “Brave Dreams” angioplasty trial found no benefit in MS but a post-hoc review found a benefit in a small proportion of the MS patients^26^.

The next site for an elevation in pressure could be within the intracranial sinuses. Sixteen percent of MS patients have a high grade stenosis of the transverse sinuses which could account for the change in sinus wall deflection^3^. However, the average transverse sinus in MS patients has been found to have a 39% effective stenosis in area and there was a 62% increase in jugular bulb height^2^, with both of these findings suggested to increase venous pressure. We are unable to estimate the change in pressure due to a change in bulb height. However, this finding almost exclusively occurs in males. As 77% of our current studies cohort are females we can estimate their pressures. The female patients had an average 48% area stenosis in the transverse sinuses but their jugular bulbs were not significantly different to the controls^2^. The hemodynamic response to a stenosis is nonlinear. Mathematical modelling of a 7.5 mm diameter cerebral vessel suggests a pressure drop across a stenosis of between 0 and 60% by area would be < 1 mmHg but a 3 mmHg pressure drop occurs with a 70% stenosis^27^. The same results hold for the venous system. In veins which vary in size from the superior vena cava to the arm veins, a 75% area stenosis was seen to correspond to a 3 mmHg pressure drop^28^. A modeling study of the sagittal sinus indicated a 38% stenosis leads to a 0.7 mmHg pressure drop^29^. Thus, at most, the average 48% stenosis in females with MS would equate to a 1 mmHg pressure drop and is probably somewhat less. As pressures which are in series are additive, the combined average increase in SSS pressure from both the neck stenoses and the transverse sinus stenoses in females would be estimated to be a maximum of 1.47 mmHg. Meaning, the transmural pressure in MS across the wall of the sinus is likely normal in the average MS patient given the 1.4 mmHg increase in ICP. Others using an indirect technique have also shown the venous pressure in MS is probably normal correlating with this finding^30^. Therefore, we would expect that a large pressure gradient would be found in a small percentage of neck veins and possibly 16% of transverse sinuses in MS and these patients may conceivably be those who may respond to angioplasty but what about the average MS patient?

If the average transmural pressure is normal, the other possibility is a change in the structure of the sinus wall. The modelling suggests either a 3.3 times increase in wall stiffness or a 49% increase in wall thickness or perhaps a combination of both. By solving Eq. (6) for both the elasticity and wall thickness (using a normal pressure) in both the MS patients and controls and calculating the combined change in these two variables by division, it can be shown that the change in elasticity multiplied by the change in wall thickness cubed is equal to 3.3. Indicating which combinations of each variable would be possible.

1 $$\Delta {\text{E}} \times \Delta {\text{D}}^{{3}} = {3}.{3}$$

In order to clarify the possibilities further we require an independent measurement. In MS the time taken for the arterial pulsation to pass into the SSS was reduced by 35% compared to controls^31^. This represents a measurement of the pulse wave velocity between the arteries and the venous system via the subarachnoid space including the spinal canal. The square of the pulse wave velocity within a vessel is equal to the elastic modulus multiplied by the wall thickness divided by the fluid density multiplied by the radius^32^ i.e.

2 $${\text{PWV}}^{{2}} = {\text{E}} \times {\text{D}}/\uprho \times {\text{R}}$$

The blood density is a constant and the difference in the sagittal sinus hydraulic diameter (proportional to the radius) in this cohort has been previously measured^2^, which would allow the radial difference to be estimated. If the dura mater of the entire system (spinal canal and sinus walls) were similarly affected, then we can solve Eq. (2) for both the controls and MS patients and by division show that.

3 $$\Delta {\text{E}} \times \Delta {\text{D}} = {2}.{7}$$

The mechanical response of the spinal canal and sinus walls have been shown to be similarly affected by MS with spinal canal pulsation propagation reduced by 40% and the venous sinus propagation by 50%^31^. Solving Eqs. (1) and (3) simultaneously gives a change in wall stiffness of 2.4 times normal and wall thickness of 1.11 times normal. Is a 2.4 fold increase in circumferential wall stiffness feasible? As previously discussed, the longitudinal stiffness is much higher (approximately 20 fold) than the circumferential stiffness in the SSS due to the longitudinal orientation of the fibers. Reorientation of the fibers into a random distribution could increase the circumferential stiffness by enough to make the findings feasible. There is chronic inflammation of the vein walls in MS. Forty seven percent of MS patients showed evidence of dural inflammation, which was equally distributed across all age groups i.e. it is likely chronic^33^. Interestingly, the lymphatics surrounding the sinus wall, together with the sinus wall itself have been shown to be an immune interface^34^, suggesting why the inflammation is centered in the sinus wall. Chronic inflammation could cause the fibers to be reoriented secondary to remodeling and scarring. There is a reduction in the type I/III collagen ratio in the jugular veins in MS^35^ and also microcalcification deposition^36^. As pointed out in the paper, an overabundance of type III collagen is associated with increased stiffness of dysfunctional bladder walls^35^ and micro calcium deposition would also be expected to increase wall stiffness similar to our findings. Finally, there is a tenfold increased risk of MS in Ehlers-Danlos syndrome (EDS) patients^37^. EDS is characterized by altered collagen synthesis and enzyme dysfunction^38^. EDS is usually thought to be associated with decreased vascular stiffness, however, one paper has suggested an increased arterial stiffness occurs compared to controls^39^. Cell culture of fibroblasts indicates that there is a significant reduction in directional fiber orientation in EDS compared to normal^40^, suggesting a random distribution of fibers in the SSS could increase circumferential stiffness but decrease the longitudinal stiffness. If an increase in the sinus wall stiffness is an important component of MS, this may shed some light into why angioplasty of apparently normal variant venous narrowing in the neck veins of MS patients may have a therapeutic effect^41^. Effectively reducing the venous pressure below normal in MS will take the otherwise stiffer sinus walls to a less stiff point in their stress/ strain curves providing a partial improvement in the physiology.

The current study was performed using MRI data which, by necessity, is acquired in the supine position. Similarly, the estimate of the normal transmural pressure was taken from the literature and was acquired in the supine position. Potentially, these two facts could limit the usefulness of the findings to other body positions. This is not an unusual limitation and is common to all studies utilizing MRI medical imaging. Despite this, the normal elastic modulus figure we found, was almost identical to that of the available literature obtained utilizing direct measurements. Our measurements were performed unblinded and there was an estimated 8% error in the linear measurements. Given an approximately 20% possible accumulated error in the methods, this compares to the 240% increase in stiffness found and therefore the error is acceptable. This is a first pass study for which the findings suggest that further investigation into the stiffness of the SSS wall is appropriate. This could be accomplished by direct measurement of samples obtained from cadaveric material similar to reference^18^.

## Methods

### Subjects

One hundred and three MS patients were prospectively recruited from an MS outpatient clinic at a tertiary referral hospital. These patients were part of a previous study^2^. There were 79 females and 24 males of average age 47.8 ± 13.0 years. There were 90 patients with relapsing remitting MS, 11 with secondary progressive MS and 2 with primary progressive MS. The clinical information regarding these patients can be found in the online data set^42^. The patients were matched to 50 previously published control patients undergoing pre-op MRI studies for stereotactic surgery for lesions thought to be unlikely to alter the intracranial pressure or compliance^3^. The surgery was for indications such as pituitary microadenoma, trigeminal artery decompression or a small meningioma less than 2 cm in size. There were 37 females and 13 males of average age 44.9 ± 10.9 years.

### Ethics approval and consent to participate

Informed consent was obtained from all patients enrolled in this study. The study was approved by the Hunter New England Area Health Ethics Committee, therefore, the study has been performed in accordance with the ethical standards laid down in the 1964 Declaration of Helsinki. The authorization number 2019ETH00912 was issued.

### MR and analysis

The patients were imaged on a 1.5 T superconducting magnet (Magnetom Avanto; Seimens, Erlangen Germany). In all MS patients, a standard brain MRI consisting of 3DT1and 3DFLAIR sagittal, T2 axial and diffusion weighted axial images was performed followed by 3DT1 post contrast imaging. All controls underwent the same pre and post contrast series, which had a 0.85 mm isotropic resolution. The MRI imaging was sourced from the hospital picture archiving and communication system (PACS) and therefore all measurements were performed on the original data.

The 3DT1 post contrast data was reformatted using the MPR software on the scanner to display the cross-section of the sinuses. A slice perpendicular to the long axis of the sagittal sinus 3 cm above the Torcular was selected. The attachment points of the two free walls of the SSS with both the falx cerebri and the inner table of the skull were defined (see Figs. 1b and 2). The length of the base of the sinus was measured for each individual. The height was measured as a line taken perpendicular to the base line centered on the vertex at the falx. The length of the free walls were calculated using Pythagoras’ theorem and designated the chord length. The triangular area of the sinus was calculated as half the base length times the height. Finally, the actual sinus area was measured by manually drawing around the sinus using the scanners measurement tool. Any sinus area below the baseline was ignored. The area of the straight sinus was measured similar to the SSS area.

The deflection of the sinus wall was estimated from the change in cross-sectional area between the actual sinus measurement and the sinus triangle area (see Fig. 2) and the chord length, using the formula for the segment of a circle:

4 $${\text{A}} \approx {2}/{3}\,{\text{s}}*{\text{h}} + {\text{h}}^{{3}} /{\text{2s}}$$

The error in this approximation is ˂ 0.1% for 0° ˂ θ ≤ 150° where θ is the angle subtended by the chord at the circle centre. A is the area between the chord and circle segment. Variable “s” is the chord length (a chord is a line which intersects a circle to produce a segment) and h is the chord height measured from the centre of the chord to the circle segment^43^. The chord height is equivalent to the sinus wall deflection. The equally distributed force can be found from the transmural pressure using the formula:

5 $${\text{F}} = {\text{P}} \times {\text{A}}$$

P is the applied transvenous pressure and A is the cross-sectional area of the upper surface of the chord^44^.

The formula for a simply supported beam relates the deflection of a beam to its mechanical properties. For a simply supported beam the following equation governs the maximum deflection produced by an equally distributed force:

6 $${\text{D}} = \left( {{5} \times {\text{F}} \times {\text{L}}^{{3}} } \right)/\left( {{384} \times {\text{E}} \times {\text{I}}} \right)$$

D is the maximum deflection which is located at the centre of the beam, F is the force, L is the length of the beam, E is the modulus of elasticity and I is the moment of inertia of cross-section^44^.

The moment of inertia of cross-section is a constant related to the geometry of the beam:

7 $${\text{I}} = \left( {{\text{W}} \times {\text{D}}^{{3}} } \right)/{12}$$

W is the beam width and D is the height of the beam. All variables are in S.I. units.

### Statistical analysis

Mean and standard deviations were obtained for each group. A Shapiro–Wilk Test was used to test for normality of the data. Differences between the groups were tested using a Mann–Whitney U test. An α ≤ 0.05 was used to assess statistical significance for all tests.

## Data Availability

All data generated or analysed during this study are included in this published article (and its Supplementary Information file).
